# Depression increases the risk of rotator cuff tear and rotator cuff repair surgery: A nationwide population-based study

**DOI:** 10.1371/journal.pone.0225778

**Published:** 2019-11-25

**Authors:** Liang-Tseng Kuo, Hong-Ming Chen, Pei-An Yu, Chi-Lung Chen, Wei-Hsiu Hsu, Yao-Hung Tsai, Ko-Jung Chen, Vincent Chin-Hung Chen

**Affiliations:** 1 Division of Sports Medicine, Department of Orthopaedic Surgery, Chang Gung Memorial Hospital, Chiayi, Taiwan; 2 College of Medicine, Chang Gung University, Taoyuan, Taiwan; 3 Department of Psychiatry, Chang Gung Memorial Hospital, Chiayi, Taiwan; 4 Health Information and Epidemiology Laboratory, Chang Gung Memorial Hospital, Chiayi, Taiwan; Indiana University, UNITED STATES

## Abstract

**Background:**

Chronic inflammation is known to be associated with both rotator cuff tears (RCTs) and depression. However, no epidemiological studies with a longitudinal follow-up have been performed to prove this association. We aimed to investigate whether depressed patients had an elevated risk of RCT and subsequent repair surgery compared with those without depression.

**Methods:**

This retrospective cohort study comprised of patients diagnosed with depression between 2000 and 2010 (depression cohort) and patients without depression (non-depression cohort, 1:2 age and sex matched). The risk of RCT and rotator cuff repair surgery were determined during a 13-year follow-up (2000–2013) between these two cohorts.

**Results:**

This study included 26,868 patients with depression and 53,736 patients without depression. The incidence of RCT was 648 and 438 per 100,000 person-years in the depression and non-depression cohorts, respectively. The adjusted hazard ratio (HR) was 1.46 (95% confidence interval [CI], 1.36–1.57) for depressed patients. The incidence of rotator cuff repair surgery was 28 and 18 per 100,000 person-years in the depression and non-depression cohorts, respectively. Depressed patients also had a significantly increased risk of subsequent rotator cuff repair surgery (adjusted HR = 1.46; 95% CI, 1.04–2.06).

**Conclusion:**

The present study showed that depression was associated with an increased risk of rotator cuff tear and rotator cuff repair surgery.

## Introduction

Recently, depressive disorders have been recognized as being associated with chronic systemic inflammation [1,2]. Patients with depressive disorders were reported to have a higher serum concentration of inflammatory markers, including interleukin (IL)-6, tumor necrosis factor (TNF)-α and IL-1β, compared with those without depressive disorders [3,4]. Depression was also found to be associated with chronic diseases related to inflammation, including chronic obstructive pulmonary disease [5,6], coronary arterial disease [7,8,9], heart failure [10], dementia [11], and arthritis [12].

Rotator cuff tear (RCT) is a common disease associated with shoulder pain and functional disability, including shoulder motion limitation and weakness [13]. The etiologies of RCT include degeneration, sports injury and trauma [14]. Some risk factors of RCT, including aging, obesity and diabetes mellitus (DM) are known to increase circulating inflammatory factors [15, 16]. In addition, chronic inflammation also contributes to a variety of age-associated diseases [17], and a number of previous studies have demonstrated its involvement in each step of tendon injury [18–20].

Although depression and RCT are both linked to chronic inflammation, epidemiological evidence for their association is lacking. Therefore, the present population-based, retrospective cohort study aimed to elucidate whether depression increases the risk of RCT, and the risk of subsequent rotator cuff surgery. The authors hypothesized that patients with depression would have an elevated risk of developing RCT and receiving subsequent rotator cuff repair surgery when compared with patients without depression.

## Materials and methods

### Study design and database

The patient data for the present retrospective, nationwide cohort study was retrieved from the Taiwan Longitudinal Health Insurance Database 2005 (LHID2005). The LHID2005 includes the claims data of 1 million insurants, who have been randomly selected from the Registry of Taiwan National Health Insurance Research Database (NHIRD), which prospectively enrolls >23 million people (almost 99% of the population of Taiwan). The de-identified data recorded in the LHID2005 includes disease diagnostic codes (as specified by the International Classification of Diseases, 9th Revision, Clinical Modification [ICD-9-CM]), date of birth, gender, residential area, income, medication prescriptions and clinical procedures. The present study was approved by Chang Gung Medical Foundation Ethics Institutional Review Board (IRB CGMH 104-7528B) and complied with their ethical standards. The committee waived the need for written informed consent.

### Patient population

**Fig 1:**
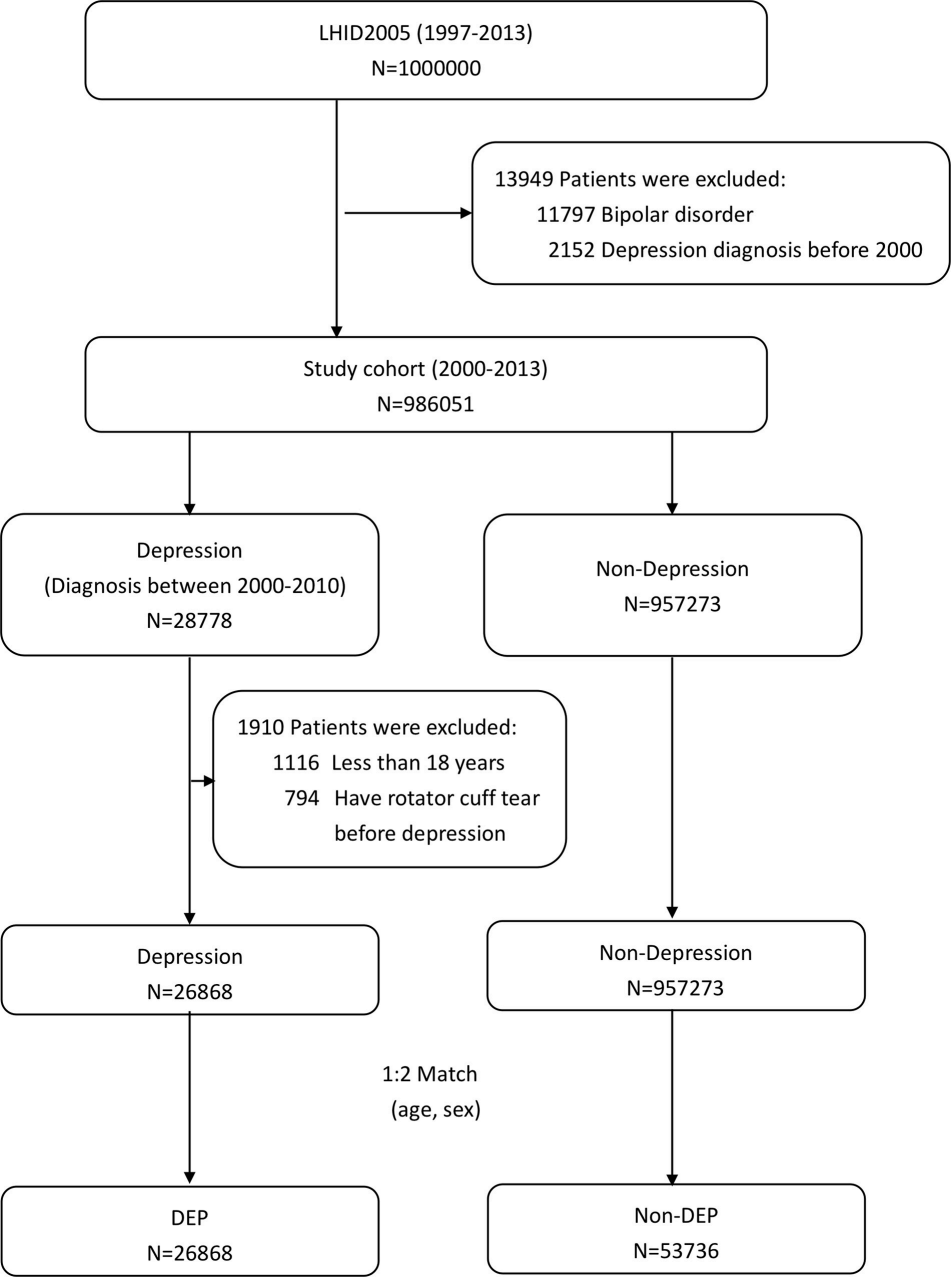
Data collection flowchart. DEP, depression; Non-DEP, non-depression; LHID2005, Taiwan Longitudinal Health Insurance Database 2005.

All patients listed in the LHID2005 with depressive disorders between January 1^st^ 1997 and December 31^st^ 2013 were identified (all ICD-9-CM diagnostic codes and National Health Insurance (NHI) procedure codes used in the present study are listed in S1 File). Only patients with depressive disorders (ICD-9-CM 296.2, 296.3, 300.4, 311) that were diagnosed by a psychiatrist between January 1^st^ 2000 and December 31^st^ 2010 were enrolled in the present study; this ensured the availability of at least three years of medical records to enable the identification of comorbidities and exclusion conditions, and at least three years of follow-up after the index date of depression diagnosis (Fig 1). The diagnosis of depression was confirmed by at least one admission claim or two following ambulatory claims; this process has been validated in a previous study [7]. The index date of diagnosis with a depressive disorder was defined as the first diagnosis recorded in the database. The follow-up period was defined from the index date of diagnosis to the date of event occurrence, withdrawal, mortality or until the end of the database (December 31^st^ 2013), whichever came first.

To enhance the diagnostic accuracy, patients who were diagnosed as having depressive disorders before January 1^st^ 2000 and who were diagnosed as having bipolar disorders were excluded. Patients younger than 18 years old and those who had a rotator cuff tendon tear before their diagnosis with depression were also excluded. The control cohort was randomly matched (1:2) from the remaining people in the LHID2005 without a history of rotator cuff tendon tear and depression by age and sex. The details of the study subject enrollment are shown in Fig 1.

### Outcomes and follow-up

The follow-up period was from January 1^st^ 2001 until December 31^st^ 2013. Any underlying comorbidities from before the index date of diagnosis were checked using the ICD-9-CM diagnostic codes. Chronic diseases, such as hypertension and DM were defined as repeated diagnosis in constitutive outpatient records when occurring within one year before the index diagnosis.

### Main outcomes

The main outcomes of this study were rotator cuff tear and rotator cuff repair surgery. The cases of rotator cuff tear were identified if they met the criteria of RCT by one admission claim or two following ambulatory claims. The diagnosis of RCT (ICD-9-CM 726.1, 727.61, 840.4) should have been determined by an orthopedic surgeon or a rheumatologist between January 1^st^ 2000 and December 31^st^ 2010 [21]. The diagnosis of RCT was confirmed by the presence of MRI (NHI procedure codes 33084B, 33085B) or ultrasonography (NHI procedure codes 19005B, 19007B) in the Taiwan NHIRD. The rotator cuff repair surgery was determined by the coexistence of a diagnosis for RCT and a rotator cuff repair surgery code (NHI procedure codes 64121B, 64122B) during hospitalization. Death was identified by a withdrawal from the NHI program [22].

### Statistical analysis

Baseline characteristics were compared between the two groups. The Chi-square test was used to analyze categorical variables. The risk of rotator cuff tendon tear and rotator cuff repair surgery between the two groups were compared using the Cox proportional hazard model, which was adjusted for all potential confounding variables listed in Table 1, except for follow-up years. The cumulative incidence rate for time to event outcomes (e.g. rotator cuff tendon tear/rotator cuff repair surgery) was plotted. A log-rank test was used to compare the survival curves of the two groups. A two-tailed *P*-value of < .05 was considered to indicate a statistically significant difference. All statistical analyses were performed using SAS version 9.4 (SAS Institute, Cary, NC, USA).

**Table 1:** Study subject demographics.

Variables	Depression (N = 26,868)		Non-depression (N = 53,736)		*P*-value
	n	%	n	%	
Gender					1.00
Male	10,318	38.4	20,636	38.4	
Female	16,550	61.6	33,100	61.6	
Age (years)					1.00
18–29	5,801	21.6	11,602	21.6	
30–39	5,798	21.6	11,596	21.6	
40–49	5,577	20.8	11,154	20.8	
50–59	4,277	15.9	8,554	15.9	
≥60	5,415	20.2	10,830	20.2	
Urbanization level					0.52
1 (City)	8,499	31.6	17,241	32.1	
2	12,303	45.8	24,550	45.7	
3	4,191	15.6	8,226	15.3	
4(Villages)	1,875	7.0	3,719	6.9	
Monthly Income (NTD)					< 0.01
0	6,777	25.2	13,321	24.8	
1–15840	5,478	20.4	8,452	15.7	
15841–25000	9,039	33.6	18,933	35.2	
>25000	5,574	20.8	13,030	24.3	
Comorbidities					
Diabetes mellitus					< 0.01
No	24,273	90.3	49,919	92.9	
Yes	2,595	9.7	3,817	7.1	
Hypertension					< 0.01
No	20,724	77.1	44,973	83.7	
Yes	6,144	22.9	8,763	16.3	
Hyperlipidemia					< 0.01
No	23,568	87.7	49,381	91.9	
Yes	3,300	12.3	4,355	8.1	
Autoimmune disease					< 0.01
No	26,486	98.6	53,284	99.2	
Yes	382	1.4	452	0.8	
Coronary heart disease					< 0.01
No	23,700	88.2	50,156	93.3	
Yes	3,168	11.8	3,580	6.7	
Cancer					< 0.01
No	25,134	93.6	51,624	96.1	
Yes	1,734	6.5	2,112	3.9	
Obesity					0.03
No	26,733	99.5	53,556	99.7	
Yes	135	0.5	180	0.3	
Gout					< 0.01
No	25,254	94.0	51,212	95.3	
Yes	1,614	6.0	2,524	4.7	
Rotator cuff tear					< 0.01
No	25,507	94.9	51,859	96.5	
Yes	1,361	5.1	1,877	3.5	
Rotator cuff tear need surgery					0.01
No	26,808	99.8	53,657	99.9	
Yes	60	0.2	79	0.1	

NTD, New Taiwan Dollar.

## Results

### Patient characteristics

Following application of the exclusion criteria, a total of 26,838 patients with depression (depression cohort) and 53,736 patients without depression (non-depression cohort) were included in the present study (Fig 1).

There were no significant differences in gender, age and urbanization level between the two cohorts (Table 1). Compared with the non-depression cohort, patients with depression had a significantly higher prevalence of comorbidities, including DM, hypertension, hyperlipidemia, autoimmune disease, coronary heart disease, cancer, obesity and gouty arthritis (Table 1). Patients with depression had a significantly higher risk of RCT and of receiving rotator cuff repair surgery compared with patients without depression (5.1% vs. 3.5%; *P* < 0.0001; Table 1). The depression group had a significantly higher risk of receiving rotator cuff repair surgery compared with the control group (0.2% vs. 0.1%; *P* < 0.05).

**Fig 2:**
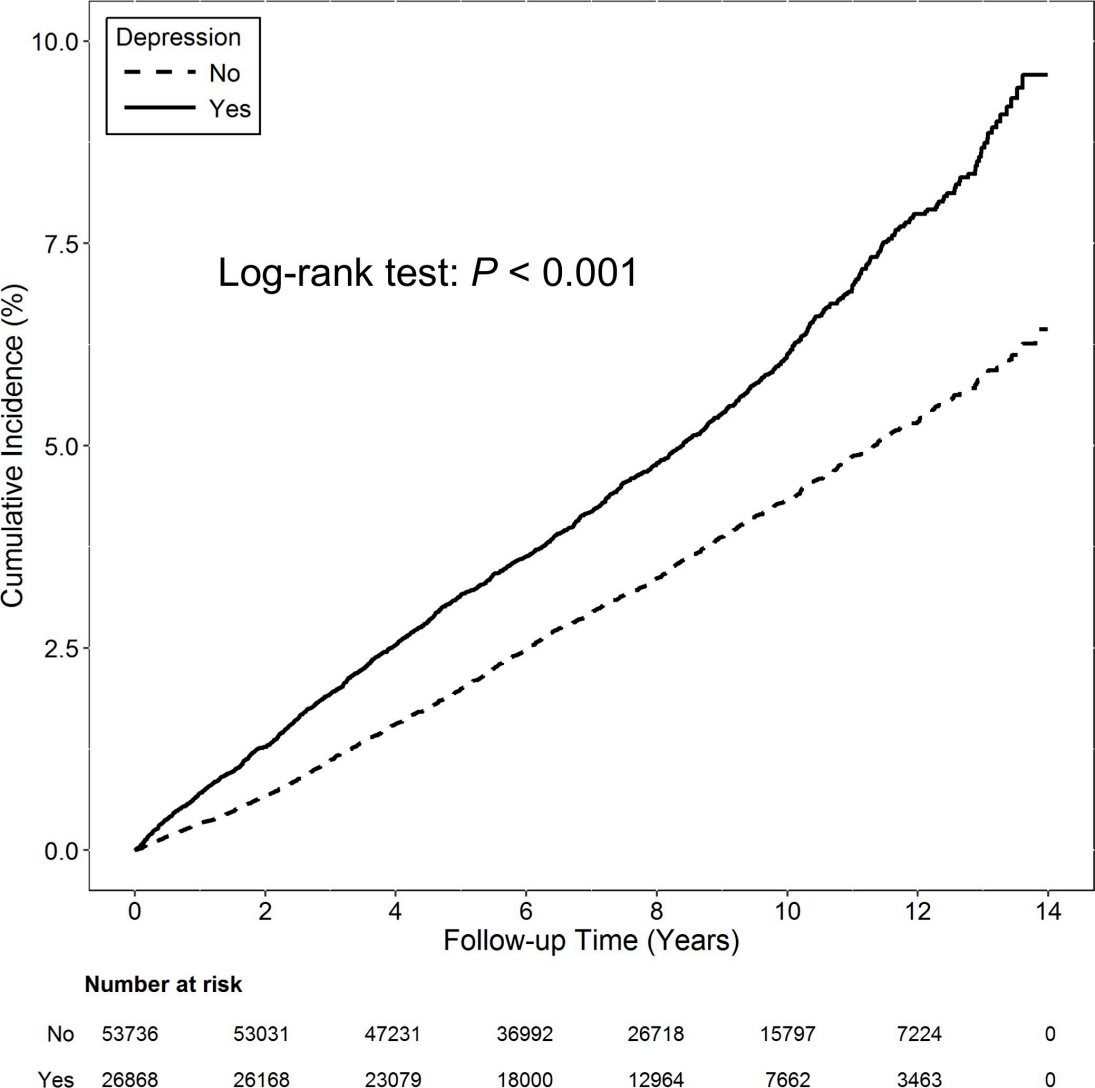
Cumulative incidence for rotator cuff tear in the depression and control cohorts during the 13-year follow-up period. The depression cohort had a significantly higher cumulative incidence of RCT than control cohort (*P* < 0.001).

**Fig 3:**
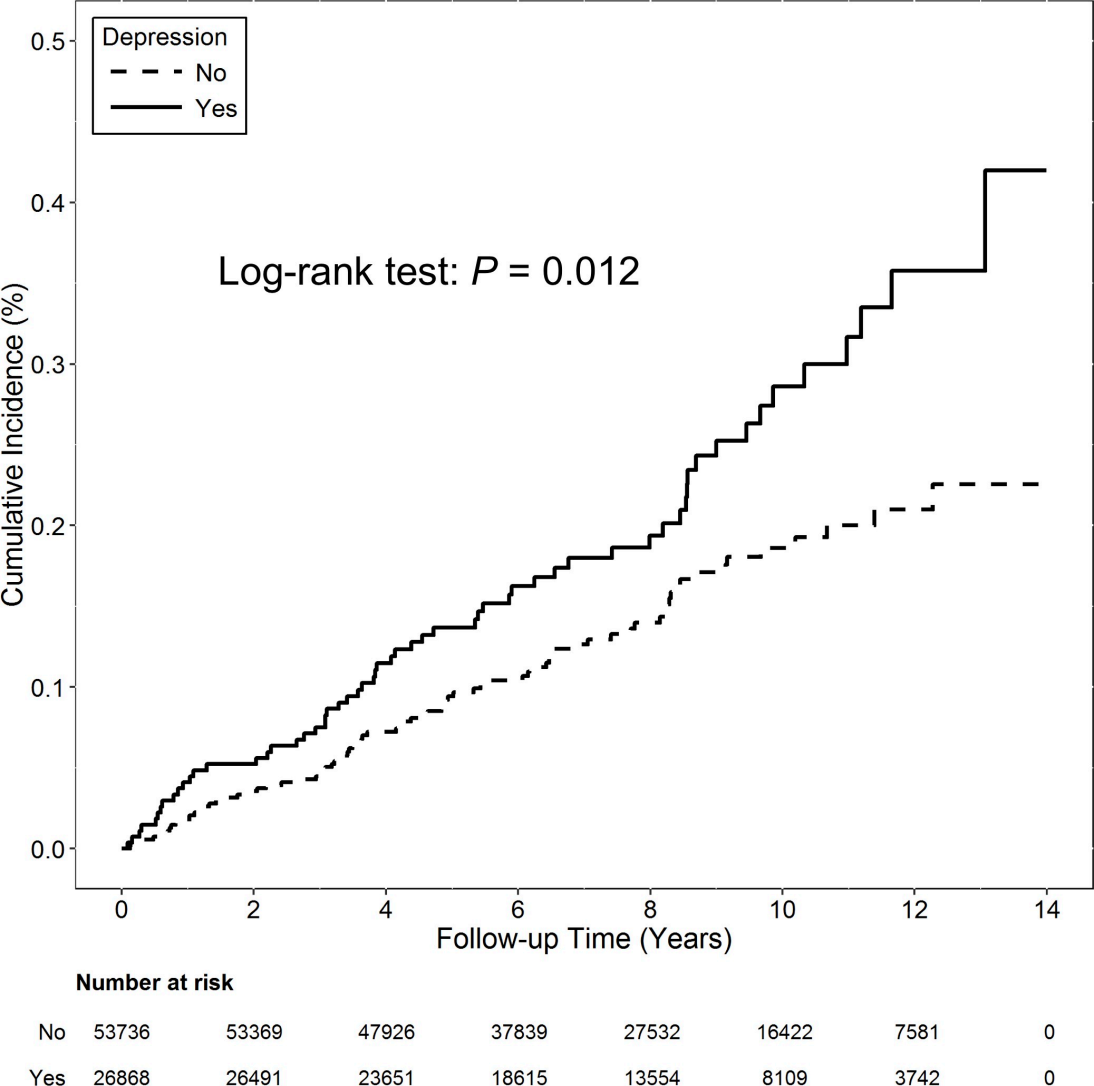
Cumulative incidence for rotator cuff repair surgery in the depression and control cohorts during the 13-year follow-up period. The depression cohort had a significantly higher cumulative incidence of rotator cuff repair surgery than control cohort (*P* = 0.012).

Compared with patients in the non-depression cohort, patients with depression had a significantly higher incidence of RCT (incidence rate: 648 vs. 438 per 100,000 person-years; *P* < 0.001; Table 2; Fig 2). Compared with patients without depression, patients with depression also had a significantly higher incidence of having subsequent rotator cuff repair surgery (incidence rate: 27.8 vs. 18.1 per 100,000 person-years; *P* = 0.012; Table 3; Fig 3).

**Table 2:** Incidence, crude and adjusted HRs, and 95% CIs for rotator cuff tear in the depression and control cohorts during the 13-year follow-up period.

Incidence of rotator cuff tear	Control Patients (n = 53,736)	Depression Patients (n = 26,868)
Events	1,877	1361
Person-years	428,743	209,938
Incidence per 100,000 person years	438	648
Crude HR (95%CI)	1.00	1.48^a^ (1.38–1.59)
Adjusted HR^b^ (95% CI)	1.00	1.46^a^ (1.36–1.57)

CI, confidence interval; HR, hazard ratio.

^a^ *P* < 0.05

^b^ Adjusted for all covariates (gender, age, urbanization, income, and comorbidities including diabetes mellitus, hypertension, hyperlipidemia, autoimmune disease, coronary heart disease, cancer, obesity, and gout).

**Table 3:** Incidence, crude and adjusted HRs, and 95% CIs for rotator cuff tear repair surgery in the depression and control cohorts during the 13-year follow-up period.

Incidence of rotator cuff tear	Control Patients (n = 53,736)	Depression Patients (n = 26,868)
Events	79	60
Person-years	436,278	215,722
Incidence per 100,000 person years	18	28
Crude HR (95%CI)	1.00	1.54^a^ (1.10–2.15)
Adjusted HR^b^ (95% CI)	1.00	1.46^a^ (1.04–2.06)

CI, confidence interval; HR, hazard ratio.

^a^ *P* < 0.05

^b^ Adjusted for all covariates (gender, age, urbanization, income, and comorbidities including diabetes mellitus, hypertension, hyperlipidemia, autoimmune disease, coronary heart disease, cancer, obesity, and gout).

### Rotator cuff tear

After adjustment for all covariates, depression was identified as an independent risk factor for rotator cuff tendon tear (hazard ratio [HR] 1.46, 95% confidence interval [CI] 1.36–1.57; Table 4). Increased age, higher urbanization and higher monthly income (> 25,000 New Taiwan Dollars) were also found to be significantly associated with a higher risk of RCTs. Other risk factors for RCT were female gender, older age and comorbidities, including DM, hyperlipidemia, autoimmune disease, coronary heart disease, obesity and gouty arthritis.

**Table 4:** Risk factors for rotator cuff tear.

Variables	Crude				Adjusted^a^			
	HR	95% CI		*P* value	HR	95% CI		*P* value
Depression								
Yes	1.48	1.38	1.59	< 0.01	1.46	1.36	1.57	< 0.01
No	1.00	Reference			1.00	Reference		
Gender								
Male	0.80	0.74	0.86	< 0.01	0.77	0.71	0.83	< 0.01
Female	1.00	Reference			1.00	Reference		
Age (years)								
18–29	1.00	Reference			1.00	Reference		
30–39	2.77	2.32	3.32	< 0.01	2.65	2.22	3.18	< 0.01
40–49	6.16	5.21	7.27	< 0.01	5.88	4.97	6.95	< 0.01
50–59	8.31	7.03	9.82	< 0.01	7.63	6.44	9.04	< 0.01
≥60	6.44	5.44	7.62	< 0.01	6.18	5.17	7.39	< 0.01
Urbanization level								
1(City)	1.16	1.00	1.35	0.05	1.25	1.07	1.46	< 0.01
2	1.11	0.96	1.29	0.17	1.19	1.02	1.38	0.02
3	1.04	0.88	1.22	0.66	1.08	0.92	1.28	0.35
4(Villages)	1.00	Reference			1.00	Reference		
Income (NTD/month)								
0	1.00	Reference			1.00	Reference		
1–15840	0.88	0.79	0.99	0.03	0.99	0.88	1.12	0.90
15841–25000	1.01	0.92	1.10	0.92	1.01	0.92	1.11	0.89
>25000	1.13	1.03	1.25	0.01	1.28	1.15	1.42	< 0.01
Comorbidities (Yes/No)								
Diabetes mellitus	1.93	1.73	2.14	< 0.01	1.16	1.03	1.30	0.02
Hypertension	1.72	1.59	1.86	< 0.01	0.90	0.81	0.99	0.03
Hyperlipidemia	2.19	1.99	2.41	< 0.01	1.31	1.17	1.47	< 0.01
Autoimmune disease	2.24	1.74	2.89	< 0.01	1.51	1.17	1.95	< 0.01
Coronary heart disease	1.94	1.75	2.15	< 0.01	1.19	1.06	1.34	< 0.01
Cancer	1.47	1.27	1.71	< 0.01	1.01	0.86	1.17	0.95
Obesity	2.62	1.78	3.85	< 0.01	1.82	1.23	2.68	< 0.01
Gout	1.69	1.47	1.93	< 0.01	1.17	1.02	1.35	0.03

NTD, New Taiwan Dollar.

^a^ Adjusted for all covariates (gender, age, urbanization, income, comorbidities).

### Rotator cuff repair surgery

After adjustment for all covariates, depression was identified as an independent risk factor for RCT repair surgery (HR 1.46; 95% CI 1.04–2.06; Table 5). Another risk factor for RCT repair surgery was coronary heart disease (HR 1.69; 95% CI 1.05–2.73; Table 5).

**Table 5:** Risk factors for rotator cuff repair surgery.

Variables	Crude				Adjusted^a^			
	HR	95% CI		*P*-value	HR	95% CI		*P*-value
Depression								
Yes	1.54	1.10	2.15	0.01	1.46	1.04	2.06	0.03
No	1.00	Reference			1.00	Reference		
Gender								
Male	0.94	0.67	1.33	0.74	0.95	0.66	1.36	0.78
Female	1.00	Reference			1.00	Reference		
Age (years)								
18–29	1.00	Reference			1.00	Reference		
30–39	6.60	1.49	29.22	0.01	6.14	1.38	27.27	0.02
40–49	17.75	4.26	73.98	< 0.01	15.44	3.69	64.57	< 0.01
50–59	30.06	7.27	124.25	< 0.01	24.47	5.87	102.00	< 0.01
≥60	30.59	7.44	125.71	< 0.01	24.33	5.76	102.89	< 0.01
Urbanization level								
1(City)	0.89	0.46	1.72	0.74	1.31	0.66	2.61	0.44
2	0.85	0.45	1.61	0.61	1.18	0.61	2.28	0.63
3	0.82	0.39	1.70	0.59	0.93	0.44	1.94	0.85
4(Villages)	1.00	Reference			1.00	Reference		
Monthly Income (NTD)								
0	1.00	Reference			1.00	Reference		
1–15840	0.62	0.34	1.15	0.13	0.71	0.38	1.32	0.27
15841–25000	1.27	0.83	1.93	0.28	1.42	0.90	2.22	0.13
>25000	0.88	0.54	1.45	0.62	1.17	0.68	2.00	0.57
Comorbidities (Yes/No)								
Diabetes mellitus	2.43	1.53	3.87	< 0.01	1.10	0.66	1.84	0.72
Hypertension	2.44	1.71	3.48	< 0.01	0.89	0.57	1.40	0.62
Hyperlipidemia	2.83	1.85	4.31	< 0.01	1.30	0.80	2.12	0.29
Autoimmune disease	2.49	0.79	7.82	0.12	1.45	0.46	4.58	0.53
Coronary heart disease	3.40	2.26	5.13	< 0.01	1.69	1.05	2.73	0.03
Cancer	2.54	1.43	4.49	< 0.01	1.52	0.85	2.71	0.16
Obesity	2.25	0.32	16.08	0.42	1.45	0.20	10.45	0.72
Gout	2.41	1.39	4.19	< 0.01	1.30	0.72	2.34	0.39

NTD, New Taiwan Dollar.

^a^ Adjusted for all covariates (gender, age, urbanization, income, comorbidities).

## Discussion

To the best of our knowledge, the present study is the first to investigate whether depression is associated with RCT and RCT repair surgery. The study revealed that patients with depression had a significantly higher risk of RCT and subsequent repair surgery compared with those without depression. After control for potential confounders, depression was shown to be associated with an increased risk of both RCT and the subsequent repair surgery. This suggests that patients with depression are at risk of developing rotator tendon injury and physicians should pay increased attention to their somatic complaints, particularly in association with their shoulders.

The underlying mechanism of how depression is associated with RCTs remains uncertain. The etiology of RCT could either be extrinsic (impingement and demographic factors) or intrinsic (degeneration, hypovascularity and inflammation) [15]. Inflammation not only plays a role in the onset and development of tendon injury but also negatively impacts the repair of injured tendons [14]. Therefore, for patients with depression, the authors suggest that the intrinsic pathogenic factors, especially inflammation, might play an essential role in the development of RCT, and therefore the subsequent repair surgery.

How do depression and the associated inflammation work together in tendon injury? Depression itself might not initiate the tear of rotator cuff tendons, however inflammation could potentiate the progression of tendinopathy and tearing [14]. Furthermore, depression could amplify the painful sensation of rotator cuff disease [23,24]. The intensity of a painful sensation can be increased by negative emotion, which may promote the diagnosis of RCT and further operation for the rotator cuff injury [23].

Depression affects both the adrenocortical axis and hormone system and can impair pain perception [24]. Patients with depression may have hyperalgesia due to the effect of pro-inflammatory cytokines, such as TNF—α, that could reduce the pain threshold both in the brain and in the dorsal root ganglia [7,25]. Similar to the present study, Lipscomb et al. reported that depressive symptoms were associated with the occurrence of upper extremity musculoskeletal symptoms in women [26]. Furthermore, we did an additional analysis to check if patients with RCT would also be risky to have depression during follow-up (S1 Fig). According to the result, patients with a diagnosis of RCT had an elevated risk of depression than those without a diagnosis of RCT (HR 1.67; 95% CI 1.55–1.81, S1 Table). That is, depression and RCT have a bidirectional association.

In addition to chronic inflammation, it was found that comorbidities, including DM, hyperlipidemia and gout may also endanger the rotator cuff tendon in patients with depression. Patients with DM had a higher risk of overall tendon rupture [27]. Ranger et al. also found that DM patients had a 3 times higher risk of tendinopathy compared with the controls [28]. For patients with gout, monosodium urate crystal can lead to inflammation, decreased function and viability of tenocytes, promoted tendon degradation and necrosis [29,30]. The released inflammatory factors and necrosis cascade can further destroy the tendon and slow down the healing of injured tendons [31]. For patients with hyperlipidemia, the deposition of xanthoma and fatty infiltration in the tendon can reduce the strength of tendons, causing increased tendon tears and decreased recovery of the ruptured tendons [32,33]. These common comorbidities could result in an elevated risk of RCT in patients with depression.

There are several strengths to the current study. To our best knowledge, it is the first large database study to confirm the association between depression and RCT and cuff repair surgery. It also limited the depression cohort to individuals who were newly diagnosed with depression, and this made the calculation of time-to-event outcomes possible. Furthermore, both RCT and RCT repair surgery outcomes were followed, and the findings of the study indicated that the severity of RCT did not affect its significant association between depression. Three limitations were encountered during the present study. First, NHIRD does not provide details of medical imaging and the functional score of disease, which did not allow for quantitative analysis of the severity of rotator cuff tendon injury. Second, the incidence of rotator cuff tendon injury might have been underestimated. NHIRD could not record alternative therapies, such as acupuncture, traditional Chinese herbs, massage and manipulation, which are usually the primary treatments for patients with shoulder pain, especially in rural areas. Third, the diagnostic accuracy of rotator cuff tendon tear may be limited, although the study setting restricted the diagnosis of RCT to specialists, and applied the use of MRI and ultrasonography as validation. Other shoulder diseases, such as adhesive capsulitis may be confused with RCT due to the overlap of symptoms.

## Conclusions

The present study is the first large-scale nation-wide retrospective cohort study illustrating the association between depression and rotator cuff diseases. Patients with depressive disorders were linked to a higher risk of RCTs and subsequent rotator cuff repair surgery. Further study is warranted to confirm whether there is a causal relationship between depression and RCTs.

## Supporting information

S1 Fig Data collection flowchart. RCT, rotator cuff tear; Non-RCT, non-rotator cuff tear; LHID2005, Taiwan Longitudinal Health Insurance Database 2005.
(TIFF)

S1 Table Risk factors for depression. (DOCX)

S1 File ICD-9-CM code used for diagnosis in the current study. (PDF)
