# Supplementary figures and images for: Depression increases the risk of rotator cuff tear and rotator cuff repair surgery: A nationwide population-based study
Source: PLoS One. 2019 Nov 25;14(11):e0225778. doi: 10.1371/journal.pone.0225778 (PMC6876882; doi:10.1371/journal.pone.0225778)

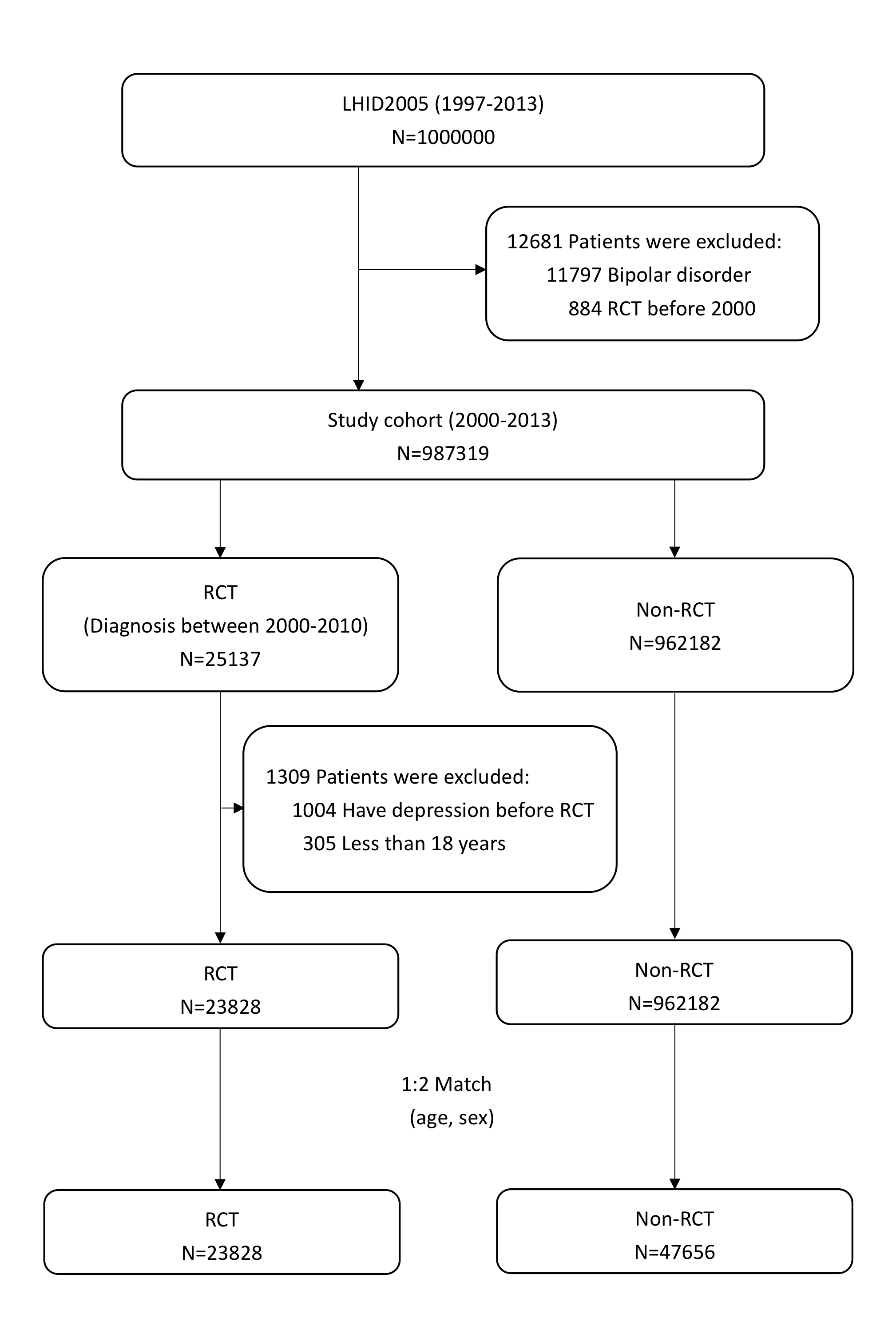

Supplement: S1 Fig — RCT, rotator cuff tear; Non-RCT, non-rotator cuff tear; LHID2005, Taiwan Longitudinal Health Insurance Database 2005. (TIFF) [file pone.0225778.s001.tiff]
