# Supplementary material for: Depression increases the risk of rotator cuff tear and rotator cuff repair surgery: A nationwide population-based study
Source: PLoS One. 2019 Nov 25;14(11):e0225778. doi: 10.1371/journal.pone.0225778 (PMC6876882; doi:10.1371/journal.pone.0225778)
Supplement: S1 Table — (DOCX) [file pone.0225778.s002.docx]

**Table S1. Risk factors for Depression.**

| Variables | Crude | | | |  | Adjusted^a^ | | | |
| --- | --- | --- | --- | --- | --- | --- | --- | --- | --- |
|  | HR | 95% CI | | *P* value |  | HR | 95% CI | | *P* value |
| RCT |  |  |  |  |  |  |  |  |  |
| Yes | 1.72 | 1.58 | 1.86 | < 0.0001 |  | 1.67 | 1.55 | 1.81 | < 0.0001 |
| No | 1.00 | Reference | |  |  | 1.00 | Reference | |  |
| Gender |  |  |  |  |  |  |  |  |  |
| Male | 0.67 | 0.61 | 0.73 | < 0.0001 |  | 0.68 | 0.62 | 0.74 | < 0.0001 |
| Female | 1.00 | Reference | |  |  | 1.00 | Reference | |  |
| Age (years) |  |  |  |  |  |  |  |  |  |
| 18-29 | 1.00 | Reference | |  |  | 1.00 | Reference | |  |
| 30-39 | 1.54 | 1.20 | 1.96 | 0.0006 |  | 1.48 | 1.16 | 1.89 | 0.0018 |
| 40-49 | 1.71 | 1.37 | 2.13 | < 0.0001 |  | 1.57 | 1.26 | 1.96 | < 0.0001 |
| 50-59 | 1.68 | 1.36 | 2.09 | < 0.0001 |  | 1.44 | 1.16 | 1.80 | 0.0011 |
| ≥60 | 1.71 | 1.38 | 2.12 | < 0.0001 |  | 1.28 | 1.02 | 1.61 | 0.0304 |
| Urbanization level |  |  |  |  |  |  |  |  |  |
| 1 (City) | 1.01 | 0.86 | 1.19 | 0.8981 |  | 0.98 | 0.83 | 1.16 | 0.8437 |
| 2 | 1.06 | 0.91 | 1.24 | 0.4617 |  | 1.03 | 0.87 | 1.21 | 0.7586 |
| 3 | 1.06 | 0.89 | 1.26 | 0.5346 |  | 1.06 | 0.89 | 1.26 | 0.5384 |
| 4 (Villages) | 1.00 | Reference | |  |  | 1.00 | Reference | |  |
| Income (NTD/month) |  |  |  |  |  |  |  |  |  |
| 0 | 1.00 | Reference | |  |  | 1.00 | Reference | |  |
| 1-15840 | 1.10 | 0.97 | 1.24 | 0.1467 |  | 1.20 | 1.06 | 1.37 | 0.0038 |
| 15841-25000 | 0.92 | 0.84 | 1.02 | 0.1274 |  | 0.94 | 0.84 | 1.05 | 0.2516 |
| >25000 | 0.83 | 0.74 | 0.93 | 0.0016 |  | 0.89 | 0.78 | 1.00 | 0.0576 |
| Comorbidities (Yes/No) |  |  |  |  |  |  |  |  |  |
| Diabetes mellitus | 1.26 | 1.12 | 1.41 | < 0.0001 |  | 1.03 | 0.90 | 1.16 | 0.7087 |
| Hypertension | 1.28 | 1.17 | 1.39 | < 0.0001 |  | 1.10 | 0.99 | 1.23 | 0.0687 |
| Hyperlipidemia | 1.42 | 1.28 | 1.58 | < 0.0001 |  | 1.21 | 1.08 | 1.37 | 0.0017 |
| Autoimmune disease | 1.45 | 1.08 | 1.94 | 0.0134 |  | 1.18 | 0.88 | 1.59 | 0.2617 |
| Coronary heart disease | 1.45 | 1.30 | 1.62 | < 0.0001 |  | 1.25 | 1.10 | 1.41 | 0.0007 |
| Cancer | 1.45 | 1.25 | 1.69 | < 0.0001 |  | 1.32 | 1.13 | 1.54 | 0.0005 |
| Obesity | 1.00 | 0.55 | 1.81 | 1.0000 |  | 0.75 | 0.42 | 1.37 | 0.3519 |
| Gout | 1.08 | 0.93 | 1.24 | 0.3302 |  | 0.98 | 0.84 | 1.14 | 0.7718 |

NTD, New Taiwan Dollar.

^a^ Adjusted for all covariates (gender, age, urbanization, income, comorbidities).
