# Supplementary material for: Depression increases the risk of rotator cuff tear and rotator cuff repair surgery: A nationwide population-based study
Source: PLoS One. 2019 Nov 25;14(11):e0225778. doi: 10.1371/journal.pone.0225778 (PMC6876882; doi:10.1371/journal.pone.0225778)
Supplement: S1 File — (PDF) [file pone.0225778.s003.pdf]

S1. ICD-9-CM code used for diagnosis in the current study.

Variable	Code
**Disease**	
Depressive disorders	296.2, 296.3, 300.4, 311
Bipolar disorder	A code: A212;<br>ICD-9-CM codes: 296.0, 296.1, 296.4, 296.5, 296.6, 296.7, 296.80, 296.89
**Main outcomes**	
Rotator cuff tear	726.1, 727.61, 840.4
Rotator cuff repair surgery	64121B, 64122B<br>(Taiwan NHI procedure code)
MRI	33084B, 33085B<br>(Taiwan NHI procedure code)
Ultrasonography	19005B, 19007B<br>(Taiwan NHI procedure code)
**Comorbidities**	
Autoimmune disease	
SLE	710.xx
Rheumatoid arthritis	714.xx
Cancer	140.xx–209.xx, 230.xx-239.xx
Diabetes mellitus	250.xx, 251.xx
Gouty arthritis	274.xx
Hypertension	401.xx–405.xx
Hyperlipidemia	272.xx
Ischemic heart disease	410.xx–413.xx, 414.0x, 414.2x, 414.8x, 414.9x, 429.2x
Obesity	278.0x
